# Imaging biomarkers for well and moderate hepatocellular carcinoma: preoperative magnetic resonance image and histopathological correlation

**DOI:** 10.1186/s12885-019-5574-8

**Published:** 2019-04-18

**Authors:** Kun Huang, Zhi Dong, Huasong Cai, Mengqi Huang, Zhenpeng Peng, Ling Xu, Yingmei Jia, Chenyu Song, Zi-Ping Li, Shi-Ting Feng

**Affiliations:** 1 Department of Radiology, The First Affiliated Hospital, Sun Yat-Sen University, 58th, The Second Zhongshan Road, Guangzhou, 510080 Guangdong China; 2 Department of Radiology, Guizhou Provincial People’s Hospital, No. 83 East, Zhongshan Road, Guiyang, 550002 Guizhou China; 3 Faculty of Medicine and Dentistry, University of Western Australia, Perth, Australia

**Keywords:** HCC, Histological grade, MRI, Gadoxetic acid

## Abstract

**Background:**

Our aim of the study is to investigate the feasibility of preoperative prediction for hepatocellular carcinoma (HCC) histological grading using gadoxetic acid-enhanced magnetic resonance imaging (MRI).

**Methods:**

This study included one hundred and fifty-six patients with solitary HCC. Preoperative gadoxetic acid-enhanced MRI findings were retrospectively analyzed. MRI qualitative features such as tumor size, margin, capsule status, signal homogeneity, intratumoral vessels, peritumoral enhancement during mid-arterial phase, peritumoral hypointensity during the hepatobiliary phase (HBP) were investigated. Apparent diffusion coefficients (ADCs), T1 reduction ratio of pre- and post-contrast enhanced images of the tumors were calculated. HCC histological grading in surgical specimens were confirmed by Edmonson’s criteria. Correlations between these MRI features and HCC histological grading were analyzed using multivariate logistic regression. The receiver operating characteristic (ROC) curve was used to assess the predictive efficacy of the model.

**Results:**

Univariate analysis showed that maximum tumor diameter (*p* = 0.004), tumor margin (*p* = 0.006), intratumoral vessels (*p* = 0.001) and peritumoral hypointensity during HBP (*p* = 0.000), were significantly correlated with HCC histological grading. There was no relationship between capsule, tumor signal, venous thrombosis, peritumoral enhancement during mid-arterial phase, ADC value, T1 reduction ratio, and HCC histological grading. Multivariate logistic regression analysis demonstrated that the maximum tumor diameter (*p* = 0.012, odds ratio = 1.002, 95% confidence interval: 1.007–1.046)) was an independent risk factor for high grade HCC.

**Conclusions:**

Greater tumor size, a more irregular margin, presence of intratumoral vessels, and peritumoral hypointensity during HBP were indicators for high grade HCC. The maximum tumor diameter was an independent risk factor for high grade HCC.

**Electronic supplementary material:**

The online version of this article (10.1186/s12885-019-5574-8) contains supplementary material, which is available to authorized users.

## Background

Hepatocellular carcinoma (HCC) is one of the most common malignancies and is associated with poor prognosis [1]. Despite the development of various treatments, including surgical resection, liver transplantation, transcatheter arterial chemoembolization (TACE), and radiofrequency ablation, postoperative recurrence remains the most important prognostic factor [2]. Some studies have reported that tumor size, number of lesions, presence of vascular invasion, and degree of tumor differentiation are important factors influencing the early recurrence of tumors [3–6]. Edmondson-Steiner grade might be an independent factor affecting prognosis or recurrence of HCC. Cucchetti, et al. [7] reported that the postoperative recurrence rate of high histological grade HCC was twice as high when compared to low grade HCC. The structure of intratumoral vessels is also related to the histological grade of HCC [8, 9].

On the other hand, preoperative histological grading of HCC is a main parameter in planning of therapeutic approach. For example, in surgical resection, the internationally recognized tumor-free margin is 1 cm around the lesion [10]. However, if the preoperative pathological grade is high, the resection range can be extended under the condition that normal liver function can be maintained post resection. According to some research, when the tumor-free margin is extended to 2 cm, the recurrence rate of HCC decreases markedly [11]. Furthermore, for postoperatively diagnosed high-grade HCCs, the follow-up period should be shortened and the frequency should be increased. More accurate inspection methods, such as dynamically enhanced magnetic resonance imaging (MRI), may also be applied to detect early recurrence or metastasis. Consequently, the role of pre-operative imaging for the assessment of well, moderate and poor differential HCC is crucial.

Preoperative pathological grading of HCC mainly relies on biopsy. However, due to the typical heterogeneity of the lesion, focal biopsy does not necessarily reflect the overall state of the tumor [12–14]. Furthermore, needle biopsy is invasive and is associated with complications such as bleeding and tumor implantation [15, 16]. Imaging methods can give an overall perspective on the tumor, which is of great significance in the preoperative evaluation of HCC. MRI is one of the most important imaging methods for liver diseases, owing to its good soft tissue resolution and multiple sequences and parameters. The application of liver-specific contrast agents has further improved the value of MRI in liver diagnosis. Gadoxetic acid is a widely applied liver-specific contrast agent. Hepatobiliary phase images obtained after gadoxetic acid enhancement not only improve lesion detection and diagnosis, but also reflect the biological behavior of a tumor at the molecular level [17, 18].

Imaging findings for HCC are closely related to the pathology of the lesions. Some studies have found that some features of contrast-enhanced ultrasound and computed tomographic (CT) images were associated with higher pathological grade of HCC [19, 20]. Heo et al. reported that the apparent diffusion coefficient (ADC) values of poorly differentiated HCCs were lower than those of well-differentiated HCCs [21]. Other studies have found that MRI enhancement patterns were closely related to the pathological grading of HCC [21–23]. However, most previous studies have focused on only one imaging characteristic, such as the tumor signal or enhancement pattern, to evaluate HCC histological grade. Few studies have comprehensively assessed the relationship between multiple imaging features and HCC histological grading [22, 24].

The purpose of this study was to analyze various qualitative and quantitative imaging features of gadoxetic acid-enhanced MRI in relation to the histological grading of single HCC lesions, and to identify the MRI features associated with high grade HCCs, in order to provide guidance for clinical treatment.

## Methods

This study received approval from the Institutional Review Board (IRB). Written informed consent was obtained from each patient.

### Patients

In this study, we retrospectively analyzed 156 HCC patients who underwent preoperative MRI examination in our hospital between November 2011 and September 2018. A total of 140 men and 16 women were enrolled, age ranged from 25 to 84 years (mean age: 53.5 ± 11.6 years). The inclusion criteria included: 1. Preoperative gadoxetic acid-enhanced MRI examination. 2. Solitary lesion without distant metastases. 3. No clinical intervention prior to MR examination and surgical resection. 4. Pathology-confirmed HCC. 5. The operation was performed within 2 weeks after MRI examination.

### MR examinations

Upper abdominal MR examination was performed on each patient using a Magnetom trio 3.0 T MR scanner (Siemens Healthcare Sector, Erlangen, Germany). Before the examination, the patients were fasted for 6 to 8 h and received adequate respiratory training. The main scan sequences and parameters include T1 weighted image (T1WI), T2 weighted image (T2WI), T1 mapping, and diffusion weighted image (DWI). The contrast agent was administrated by an automatic injector at a rate of 2 mL/s (0.025 mmol/kg). The images of gadoxetic acid-enhanced arterial phase, portal venous phase, equilibrium phase and hepatobiliary phase were acquired at 10–40 s, 50–80 s, 90–120 s and 20 min after contrast agent administration, respectively.

### Image analysis

Two experienced radiologists blinded to the pathological results evaluated all MR images independently. Qualitative MRI features were confirmed by the consistency of their reports. To avoid the possible measurement errors in inter-reader, the final results of quantitative MRI characteristics were acquired by the average of both radiologists.

Quantitative MRI features were as followed: 1. The maximum diameter of the tumor; 2. Apparent diffusion coefficient (ADC); 3. Percentage decrease in T1 (T1_D_%). The maximum diameters were measured in axial and coronal images of hepatobiliary phase. T1 relaxation times on a plain scan (T1_P_) and on the hepatocellular phase (T1_E_) were measured in the solid component of the tumor, avoiding visible vessels and necrosis, by regions of interest (ROIs) as large as possible. T1_D%_ was calculated by the following equation: T1_D%_ = (T1_P_ - T1_E_)/ T1_P_ × 100% [25, 26].

The following qualitative MRI features were evaluated: tumor thrombus, signal intensity, tumor capsule, tumor margins, intratumoral vascular, peritumoral enhancement, and peritumoral hypointensity. These were defined as follows [25, 26]:

1. Tumor thrombus: filling defect in the portal or hepatic vein with similar enhancement pattern of HCC lesion.
2. Signal intensity: homogeneous or heterogenous signal intensity of the tumor on T2WI.
3. Tumor capsule: Tumors were divided into three groups according to the status of tumor capsule: complete capsular, incomplete capsular and non-capsular tumor.
4. Tumor margins: Tumors were divided into four groups according to the forms evaluated on the hepatobiliary phase image: infiltrating lesion, smooth nodules, protruding nodules, and fusion of multiple nodules.
5. Intratumoral vessels: visible tumoral vessels on the arterial images.
6. Peritumoral enhancement: dominant enhanced around the tumor on the arterial image with isointensity on the portal and equilibrium images.
7. Peritumoral hypointensity: hypointensity around the tumor on the hepatobiliary images. The signal intensity is slightly higher than that of the tumor and lower than that of the surrounding normal liver parenchyma.

### Pathological analysis

Gross specimens of postoperative HCC were drawn and sliced continuously, with slices ranging from 0.3 cm to 2.0 cm depending on the size of the lesion. The specimens were routinely prepared with 4% formaldehyde, decalcified using 8% hydrochloric acid, embedded in paraffin, and sliced for hematoxylin and eosin staining. Multiple samples were collected in each lesion. The specimens were examined using a light microscope by experienced pathologists blinded to the MRI results. Eight slices of each lesion were analyzed, and the histological grading of each slice was evaluated, with the highest grading used as the overall result. All the specimens were graded using the Edmonson method. According to the differentiation degree of tumor cells, HCC were categorized into grades I to IV. Grades I and II were defined as low-grade HCC, grade III was defined as medium-grade HCC, and grade IV was defined as high-grade HCC.

### Statistical analysis

The statistical analysis was performed using SPSS 20 software. Tumor histological grade was used as the dependent variable, and the MR image features were used as independent variables. In the univariate analysis, categorical data were compared using chi-square tests or Fisher’s exact tests. Results with *P* < 0.05 were considered statistically significant. For the multivariate analysis, variables with significant differences in the univariate analysis were included in a stepwise logistic regression model. The forward (LR) method was used, with inclusion criteria of 0.05 and exclusion of 0.1. Results with *P* < 0.05 were considered to indicate significant effects. Receiver operating characteristic (ROC) analyses were performed for quantitative variables, with the histological grade of HCC taken as the “gold standard.”

## Results

### Histological grade

Based on the Edmonson grading method, the cohort of patients included 8 cases with grade I HCC, 104 cases with grade II, 44 cases with grade III, and 0 cases with grade IV. These cases were recategorized into low-grade HCC (112 cases), medium-grade HCC (44 cases), and high-grade HCC (0 cases).

### Qualitative MRI features

Tumor thrombi were detected in 11 patients (7%). Homogeneous tumor signal intensity was found in 57 cases (36%). There were 55 complete capsular tumors (35%), 86 incomplete capsular tumors (55%), and 15 non- capsular tumors (9%). There were 39 tumors of smooth solitary nodules (25%), 77 protruding nodules (49%), and 40 cases of fusion of multiple nodules (26%). Intratumoral vessels were detected in 67 cases (43%). Peritumoral enhancement was found in 40 cases (26%), and peritumoral hypointensity was observed in 40 cases (26%).

### Quantitative MRI features

The maximum diameters of the 156 HCCs ranged from 11.5 mm to 183.0 mm, and the average diameter was 48.9 ± 32.1 mm. DWI sequence was performed in 126 patients, and the ADC values were (0.24–2.16) × 10^− 3^ mm^2^/s, with the average value of (0.98 ± 0.26) × 10^− 3^ mm^2^/s. T1 mapping was performed in 97 patients. And the calculated T1_D%_ values were 10–90%, with the average value of 36 ± 13%.

### Correlation analysis

**Fig. 1:**
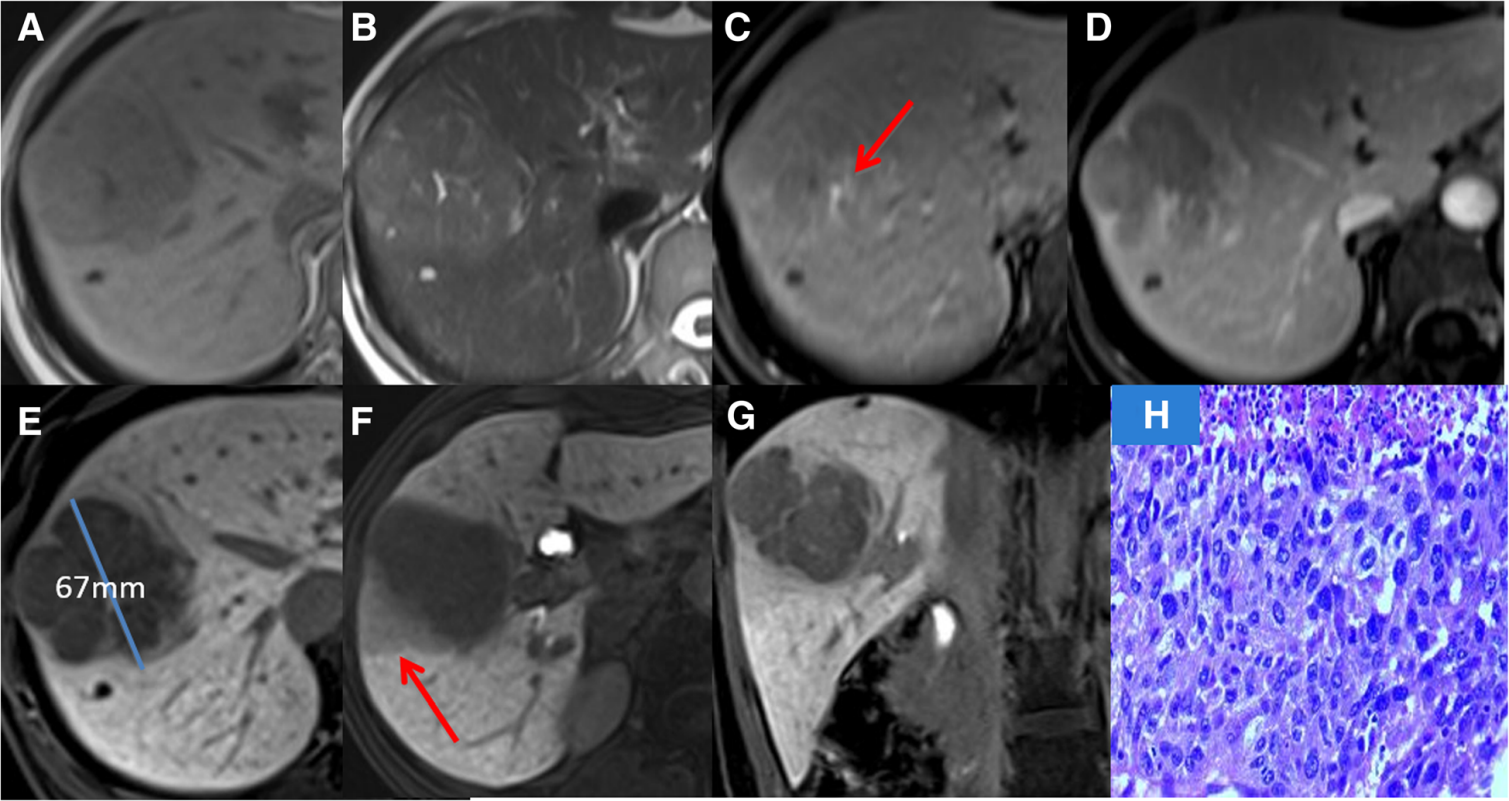
Patient with hepatocellular carcinoma (HCC) in his/her forties. T1WI (**a**) and T2WI (**b**) images showing heterogeneous signals in the tumor. Intratumoral vessels are visible on the arterial phase (**c**, arrow), and the delayed phase shows an incomplete tumor capsule (**d**). The tumor was of multinodular type with maximum diameter 67 mm, and peritumoral hypointensity (arrow) is seen on the hepatobiliary phase (**e**-**g**). The pathologic result of hematoxylin and eosin staining of a tumor tissue section (**h**) was medium-differentiated HCC

**Fig. 2:**
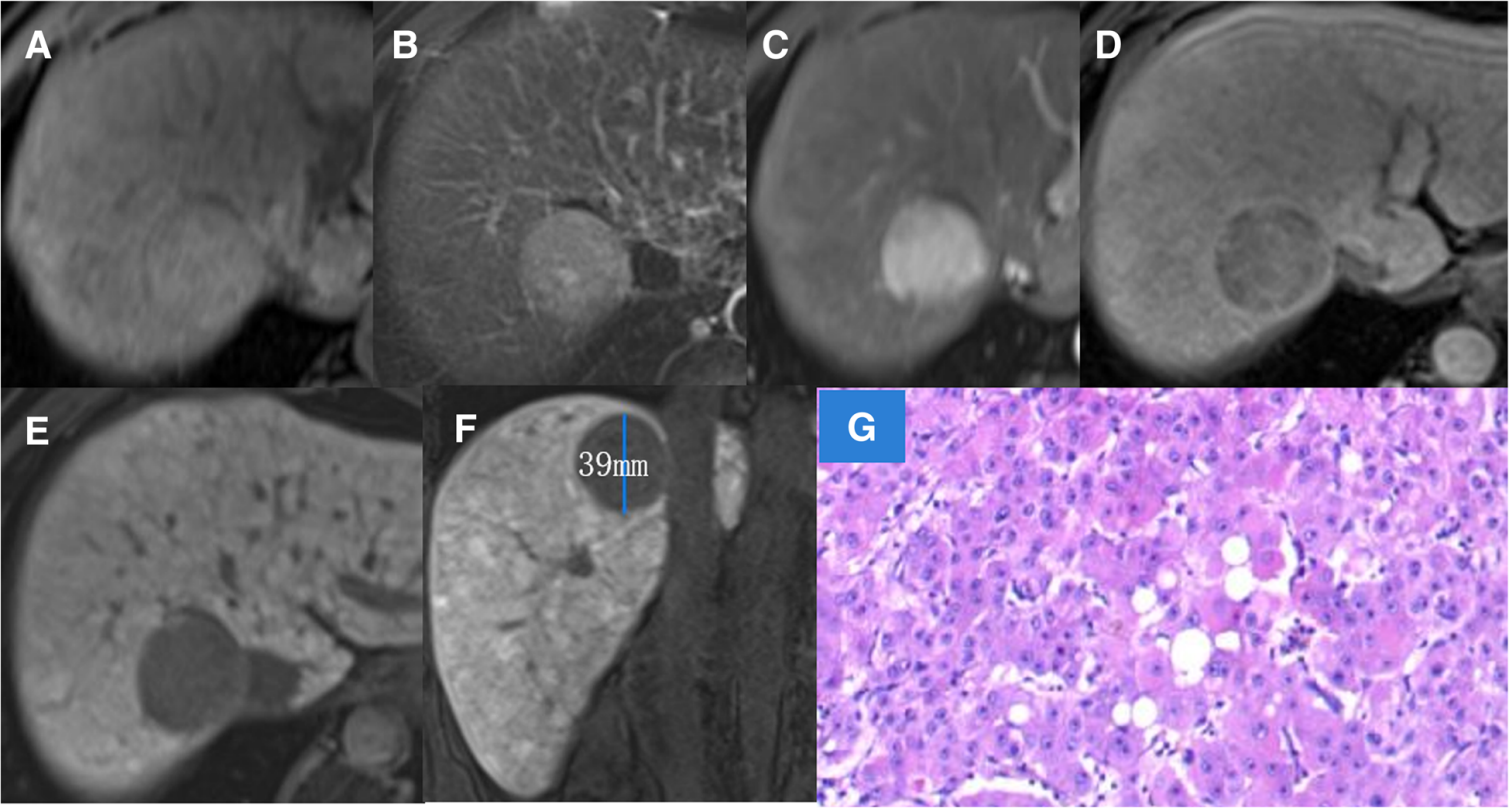
Another patient with hepatocellular carcinoma (HCC) in his/her forties. T1WI (**a**) and T2WI (**b**) images showing homogeneous signal of the tumor. No intratumoral vessels are visible on the arterial phase (**c**), and the delayed phase shows a complete tumor capsule (**d**). The tumor was a smooth nodule with maximum diameter 39 mm, and there was no peritumoral hypointensity on the hepatobiliary phase (**e**, **f**). The pathologic result of hematoxylin and eosin staining of a tumor tissue section (**g**) was highly-differentiated HCC

According to the univariate analysis, there was statistical significance in predicting HCC histological grade for the tumor margin type (*χ*^2^ = 5.305, *P* = 0.006). A pairwise comparison demonstrated that the mean HCC histological grade of single nodular tumors was lower than multinodular fusion tumors (*P* = 0.002). There was no significant difference identified between the single-nodular type and the locally protruding type (*P* = 0.940), as well as between locally protruding nodules type and multinodular fusion type (*P* = 0.117) There were statistically significant differences between HCCs with and without intratumoral vascularity (*χ*^2^ =7.412, *p* = 0.001). The histological grade of HCCs with intratumoral vascularity tended to be higher than that of HCCs without intratumoral vascularity. HCCs with and without peritumoral hypointensity also showed statistically significant differences (*χ*^2^ = 8.722, *p* = 0.000). HCCs with peritumoral hypointensity had higher histological grades (Figs. 1, 2). There was no statistically significant relationship between HCC histological grade and the other qualitative MRI features, including tumor thrombus, signal intensity, tumor capsule, intratumoral vascular, and peritumoral enhancement. (Table 1).

**Table 1:** Univariate analysis comparing qualitative magnetic resonance imaging (MRI) features and hepatocellular carcinoma (HCC) differentiation level

Imaging features		Low-grade	Medium- grade	*χ* ^2^	*P*
Tumor thrombus	Yes	5(45%)	6(55%)	2.117	0.117
	No	107(74%)	38(26%)		
Signal intensity	homogeneous	44(77%)	13(23%)	0.852	0.428
	heterogeneous	68(68%)	31(31%)		
Tumor capsule	Complete	45(82%)	10(18%)	2.311	0.103
	Incomplete	55(64%)	31(36%)		
	None	12(80%)	3(20%)		
Tumor margins	smooth solitary nodule	33(85%)	6(15%)	5.305	0.006
	protruding nodule	58(75%)	19(25%)		
	fusion of multiple nodules	21(53%)	19(47%)		
Intratumoral vascularity	s	42(58%)	31(42%)	7.412	0.001
	No	70(84%)	13(16%)		
Peritumoral enhancement	Yes	24(60%)	16(40%)	2.042	0.133
	No	88(76%)	28(24%)		
Peritumoral hypointense	Yes	20(49%)	21(51%)	8.722	0.000
	No	92(80%)	23(20%)		

The average value of maximum tumor diameter in the low-grade tumor group was 43.61 mm, and the average value of maximum tumor diameter in the medium-grade group was 62.36 mm. The maximum tumor diameter of the medium-grade group was significantly higher than the low-grade group (t = − 2.611, *p* = 0.004). The average ADC value for the low-grade group was 1.01 × 10^− 3^ mm^2^/s, and the average ADC value for the medium-grade group was 0.95 × 10^− 3^ mm^2^/s. There was no significant difference between these two groups (t = 0.659, *p* = 0.519). The average T1_D%_ in the low-grade group was 36%, and the average T1_D%_ in the medium-grade group was 33%. No significant difference was found between these two groups (t = 0.838, *p* = 0.436) (Table 2). The inter-reader agreement of in these quantitative MR image features was shown in Additional file 1.

**Table 2:** Correlation analysis for quantitative magnetic resonance imaging (MRI) features and hepatocellular carcinoma (HCC) differentiation level

	Low-grade	Medium- grade	*t*	*p*
Maximum diameter (mm)	43.61 ± 27.79	62.36 ± 38.12	−2.611	0.004
ADC (×10^−3^ mm^2^/s)^a^	1.01 ± 0.27	0.95 ± 0.23	0.659	0.519
T1_D_%^a^	36 ± 12	33 ± 15	0.838	0.436

^a^*ADC* apparent diffusion coefficient, *T1*_*D%*_ percentage decrease in T1

**Table 3:** Receiver operating characteristic (ROC) analysis of tumor maximum diameter for predicting the degree of differentiation of hepatocellular carcinoma

variable	AUC	*P* value	sensitivity	specificity	OR	95% C.I. for OR
maximum diameter (mm)	0.666	0.001	47.7%	83%	0.361	0.571–0.762

*AUC* area under the ROC curve, *OR* odds ratio, *C.I.* confidence interval

### Multivariate analysis of HCC histological grade and MRI features

The maximum diameter of the tumor was an independent predictor of HCC with high histological grade (*p* = 0.012, OR = 1.002, 95% CI: 1.007–1.046). However, tumor margins (*p* = 0.230), intratumoral vascularity (*P* = 0.432), and peritumoral hypointensity (*p* = 0.178) were not independent predictors of HCC histological grading.

**Fig. 3:**
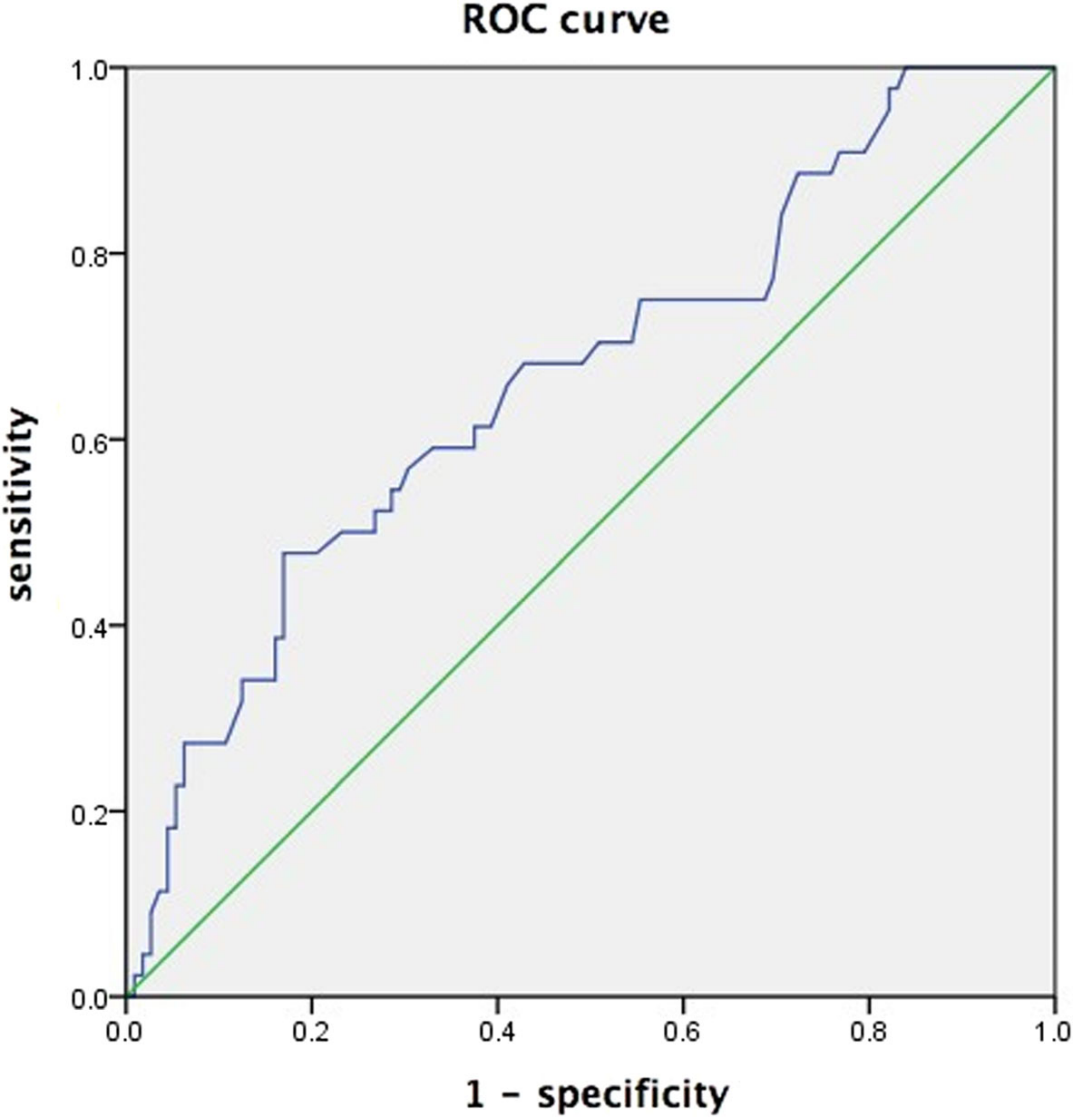
Receiver operating characteristic (ROC) curve of prognostic accuracy of HCC differentiation by maximum tumor diameter

The prediction accuracy was analyzed in terms of a ROC curve, using the histological grade of HCC as gold standard and the maximum diameter of the tumor as predictor. (Table 3, Fig. 3) The critical value for prediction was 53 mm, and the area under the ROC curve was 0.666. Using the critical value as a threshold, the odds ratio for predicting the histological grade was 0.361, with 95% confidence interval 0.571–0.762.

## Discussion

This study found that the tumor maximum diameter was an independent predictor of HCC histological grading. Other imaging features, including irregular tumor margin, presence of intratumoral vessels and peritumoral hypointensity were also associated with the higher histological grading of HCC. Other qualitative MRI features such as tumor thrombus, signal intensity, tumor capsule, tumor margins, and peritumoral enhancement, as well as quantitative features including ADC value and percentage decrease in T1 (T1_D_%), had no statistical correlation with HCC histological grades.

Tumor maximum diameter is an important prognostic factor for HCC. With each increase in tumor size, HCCs become more prone to blood vessel invasion and extrahepatic metastasis, which are associated with higher histological grades [27, 28]. According to Lee et al., 86% of medium-grade HCCs are more than 20 mm in diameter, compared to 52% of low-grade HCCs [24]. Our results also demonstrated that the maximum diameters of tumors in medium-grade HCC were larger than those in low-grade HCC. A total of 79% of the HCCs with maximum diameter less than 53 mm were low-grade HCCs, while 51% of HCCs with diameters more than 53 mm were medium-grade. Similarly, Pawlik et al. also found that the HCCs with diameter greater than 50 mm had higher histological grades [29].

The univariate analysis showed that imaging features including tumor margin, intratumoral vessels, and peritumoral hypointensity were related to HCC histological grading. However, the multivariate analysis found that these characteristics are not independent risk factors for HCC. This outcome may be a result of interaction among these features in the multivariate analysis. According to the classification method of the Japanese liver cancer research team, the edge of the tumor can be categorized into five forms [30]. As did Chou’s study [31], our study included three of these categories, the single nodule, focal protruding nodule, and multinodular fusion types. We found that multinodular fusion HCC tends to have higher histological grade compared with focal protruding nodular and single-nodular HCC. Another study found that most HCCs with irregular margins were moderately or poorly differentiated, and that the sensitivity and specificity were 82.4 and 47.6%, respectively [24]. On the other hand, a different study reported that there was no association between the tumor margin and the histological grade of HCC [22]. However, in that study, tumor margin evaluation was performed on conventional gadoxetic acid-enhanced images. Therefore, the results may have been biased by factors such as peritumoral enhancement. In comparison, gadoxetic acid is a hepatobiliary-specific MRI contrast agent. After intravenous injection, most HCCs is characterized as an area of relatively low signal without gadoxetic acid uptake during hepatobiliary phase, while the surrounding normal liver parenchyma present high signal because of their high uptake ability for gadoxetic acid. Hence, the difference in signal between HCC and surrounding liver parenchyma becomes more significantly during the hepatobiliary phase of gadoxetic acid enhanced MRI than conventional contrast agents. This makes the boundaries of tumors clearer to delineate where a more acurate evaluation of tumor margin becomes possible [32].

Presence of intratumoral vessels are an important feature for the diagnosis of HCC. The positive predictive value for HCC is 90% when a liver lesion demonstrates visible intratumoral vessels, with a specificity of 98% [33]. According to Lee et al. [24], about 65% of high-grade HCCs were found to contain intratumoral vessels, and about 64% of medium-grade HCCs were associated with intratumoral vessels; however, intratumoral vessels were absent in 71% of low-grade HCCs [24]. Consistent with the results of Lee’s [24] study, our results showed that HCCs with intratumoral vessels tended to have higher histological grades than those without intratumoral vessels. In our study, 70% of medium-grade HCCs were found to contain intratumoral vessels, but only 38% of low-grade HCCs.

Peritumoral hypointensity refers to an area around the tumor, which is hyperintense to the tumor tissue but hypointense to the normal hepatic parenchyma during the hepatobiliary phase [34]. In this study, we found that 48% of medium-grade HCCs showed hypointensity around the tumor, but only 18% of low-grade HCCs. According to previous reports, occlusion of micro-portal veins caused by microvascular tumor thrombus and insufficiency of the arterial blood supply result in the dysfunction of local liver cells, which subsequently leads to decreases in the uptake of gadoxetic acid, resulting hypointensity around the tumor during the hepatobiliary phase [35–37]. Therefore, we speculate that the peritumoral hypointensity of HCC with high histological grade is associated with microvascular invasion.

Many studies have shown that vascular invasion occurs often in high-grade HCC [38–41]. Vascular invasion can be divided into microvascular invasion and macrovascular invasion [42]. Currently, microvascular invasion cannot be directly visualized on imaging [43]. The venous tumor thrombus in this study only involved large veins, including large branches of the portal vein and hepatic vein. Although many previous studies have shown that vascular invasion is closely related to the level of tumor tissue differentiation [38–41], this study did not find a relationship between venous tumor thrombi visible on MRI and the histological grade of HCC. This may be a result of selection bias in the study sample, and a larger sample size is required for further validation.

Witjes et al. [22] reported no differences in the incidence of heterogeneity on T2WI among different levels of HCC differentiation. Our study also found no correlation. However, Lee et al. [24] found that HCC with heterogeneity tended to have poor differentiation and high histological grade, and that medium- or high-grade HCCs were more susceptible to necrosis, resulting in heterogenous density on CT images.

In this study, equilibrium phase images were used to evaluate the existence and integrity of a tumor capsule, which is defined as a hyperintense ring around the lesion in delayed or equilibrium phase contrast enhanced MRI [44]. The delayed enhancement of the tumor capsule is associated with slower blood flow in micro-vessels [44, 45]. However, not all HCCs demonstrate tumor capsules: previous studies suggest 24–90% of Asian HCC patients, and 12–42% of non-Asian HCC patients demonstrates tumor capsules [46–50]. Some researchers have suggested that a tumor capsule is one of the characteristics of high-grade HCC [48, 49]. In this study, there was no correlation between the tumor capsule status on contrast-enhanced MRI and the histological grade, which is consistent with the results of Lee’s [24] study. Possible explanations are that the degree of HCC differentiation has relatively little effect on the tumor capsule, or that the tumor capsule of some lesions is very thin and may not be observed on MRI.

Although the mechanisms of peritumoral enhancement are not fully understood, most researchers believe that it may be caused by microvascular invasion, which causes occlusion of the microportal veins and a compensatory increase in hepatic arterial blood supply [51]. Studies have shown that high-grade HCC is prone to microvascular invasion [52, 53]. In this study, however, no correlation was found between peritumoral enhancement and HCC histological grade. This may be related to the limited sensitivity of MRI to peritumoral enhancement.

ADC value had been use to assess grade of HCC in previous studies have. Heo et al. [21] found that the ADC value and the degree of HCC differentiation were negatively correlated. Nakanishi et al. [54] reported that the ADC value in high-grade HCC was significantly lower than in medium- and low-grade HCC [55]. However, the results of our study demonstrated no statistical relationship between HCC grade and ADC values. The difference may be due to the relative larger lesions in our research which represent higher degree of degeneration and necrosis. At the same time, not all the patients in the study were received DWI. Therefore, selective bias could not be fully avoided. On the other hand, the instability of ADC value might be another limitating factor. The determination of the ADC value is easily influenced by many factors, such as differences in the heartbeat and respiratory control of patients, due to the intrinsical sensitivity of echo echoplanar imaging (EPI) sequence to motion artifacts [55].

Peng et al. [56] showed that the percentage of T1 reduction was different before and after gadoxetic acid-enhancement in HCCs with different pathological grades. However, our results showed no correlation between the percentage of T1 reduction and the histological grade of HCC. This difference may be a result of selection bias in the samples. The study of Peng included 53 HCC lesions, all of which included T1 mapping images, and among which 13 lesions were pathologically diagnosed as grade I. In this study, only 97 cases included T1 mapping images, and only 8 cases were diagnosed as grade I HCC. In this study, we combined grade I and grade II HCC together as low-grade group, and grade III as medium grade group, to evaluate the difference of T1_D_% between low and medium grade group. Because there were only 8 cases of grade I HCC, the result of this study is also equivalent to a finding that the T1_D_% of grades II and III showed no statistically significant difference, which is similar to the results of Peng’s study. However, the study sample was relatively small and further study is needed to confirm the role of T1 mapping for prediction of HCC grading.

In this study, there were several imaging features (tumor margin, intratumoral vessels, peritumoral hypointensity, and maximum tumor diameter) which demonstrated statistically significant correlation with the histological grade of HCC during univariate logistic analysis. This will be due to the failure of adjustment the interaction effect of the factors for univariate logistic analysis. When many factors were excluded from each other in multivariate logistic regression analysis, only the maximum tumor diameter was an independent risk factor for high grade HCC. Tumor diameter has been included in liver cancer staging systems, such as the TNM staging system and Milan liver transplantation criteria [57, 58]. Consequently, tumor diameter has been showed as the most significant influence on the biological behaviors, such as grading, microvascular invasion, recurrence or prognosis in HCC.

There are some limitations in this study. First, most of the HCC lesions in this study were large nodules or masses, the results of this study may not be applicable for small HCCs. Second, the cohort in this study is heterogeneous because mostly consisted of grade 2 and grade 3 HCCs, with only a few grade 1 HCCs and no grade 4 HCC.. Third, some of the patients in the study had not the quantitative MRI features (ADC value, T1 _D_%). Thus, the selective bias could not be fully avoided.

## Conclusion

In conclusion, greater size, a more irregular margin, presence of intratumoral vessels, and peritumoral hypointensity during HBP indicate a higher histological grade of HCC. The maximum tumor diameter is an independent risk factor for high grade HCC. Our study has demonstrated that the radiological prediction of HCC grades is possible before treatment is practicable which can help determine the most appropriate surgical strategies for patients based on more accurate risk-benefit assessment. .

## Additional file

Additional file 1: Interobservers agreement in quantitative MR image features. This table shows the interobservers agreement in quantitative MR image features measured by two radiologists. (DOCX 15 kb)

## Abbreviations

ADC: Apparent diffusion coefficient
CT: Computed tomography
DWI: Diffusion weighted image
HASTE: Half-Fourier single shot turbo spin-echo
HCC: Hepatocellular carcinoma
IRB: Institutional Review Board
MRI: Magnetic resonance imaging
ROC: Receiver operating characteristic
T1WI: T1 weighted image
T2WI: T2 weighted image
TACE: Transcatheter arterial chemoembolization
