# Supplementary material for: Imaging biomarkers for well and moderate hepatocellular carcinoma: preoperative magnetic resonance image and histopathological correlation
Source: BMC Cancer. 2019 Apr 18;19:364. doi: 10.1186/s12885-019-5574-8 (PMC6472074; doi:10.1186/s12885-019-5574-8)
Supplement: Supplementary file 1 — Interobservers agreement in quantitative MR image features. This table shows the interobservers agreement in quantitative MR image features measured by two radiologists. (DOCX 15 kb) [file 12885_2019_5574_MOESM1_ESM.docx]

Supplementary Table Interobservers agreement in quantitative MR image features.

| MRI features (91 cases) | Observer A  (15 years experience) | Observer B  (3 years experience) | Agreement test  (kappa value) |
| --- | --- | --- | --- |
| Maximum diameter (mm) | 48.89±32.02 | 48.97±32.17 | 0.373 |
| ADC (×10^-3^ mm^2^/s)* | 0.98±0.26 | 0.98±0.26 | 0.243 |
| T1_P_ | 1200.73±428.34 | 1201.27±428.51 | 0.204 |
| T1_E_ | 752.28±271.16 | 753.12±271.07 | 0.193 |

T1_P_: T1 relaxation times on a plain scan; T1_E_: T1 relaxation times at the hepatocellular phase.
